# Milk Odd and Branched Chain Fatty Acids in Dairy Cows: A Review on Dietary Factors and Its Consequences on Human Health

**DOI:** 10.3390/ani11113210

**Published:** 2021-11-10

**Authors:** Sidi Ka Amar Abdoul-Aziz, Yangdong Zhang, Jiaqi Wang

**Affiliations:** State Key Laboratory of Animal Nutrition, Institute of Animal Sciences, Chinese Academy of Agricultural Sciences, No. 2 Yuanmingyuan West Road, Beijing 100193, China; sidikaaziz86@gmail.com (S.K.A.A.-A.); zhangyangdong@caas.cn (Y.Z.)

**Keywords:** milk, odd, branched, fatty, acids, cows, dietary

## Abstract

**Simple Summary:**

The objective of this review is to highlight the importance of odd and branched chain fatty acids and dietary factors that may affect their content in milk acids in dairy cows. The primary source of odd and branched chain fatty acids is ruminal bacteria. In contrast to saturated fatty acids, odd and branched chain fatty acids have health protective effects against certain diseases as cardiovascular diseases, type II diabetes, cancers, Alzheimer’s disease and metabolic syndrome. Ruminant products are the main source of these fatty acids in the human diet. Odd and branched chain fatty acids profile in cow milk is mainly affected by dietary fatty acids and fatty acids metabolism in the rumen. Additionally, lipid mobilization in the body and fatty acids metabolism in mammary glands affect the milk odd and branched chain fatty acids profile. Understanding the origin of odd and branched chain fatty acids in milk and manipulating the diet of dairy cows to produce odd and branched chain fatty acids-enriched milk can be of scientific and industrial significance.

**Abstract:**

This review highlights the importance of odd and branched chain fatty acids (OBCFAs) and dietary factors that may affect the content of milk OBCFAs in dairy cows. Historically, OBCFAs in cow milk had little significance due to their low concentrations compared to other milk fatty acids (FAs). The primary source of OBCFAs is ruminal bacteria. In general, FAs and OBCFAs profile in milk is mainly affected by dietary FAs and FAs metabolism in the rumen. Additionally, lipid mobilization in the body and FAs metabolism in mammary glands affect the milk OBCFAs profile. In cows, supplementation with fat rich in linoleic acid and α-linolenic acid decrease milk OBCFAs content, whereas supplementation with marine algae or fish oil increase milk OBCFAs content. Feeding more forage rather than concentrate increases the yield of some OBCFAs in milk. A high grass silage rate in the diet may increase milk total OBCFAs. In contrast to saturated FAs, OBCFAs have beneficial effects on cardiovascular diseases and type II diabetes. Furthermore, OBCFAs may have anti-cancer properties and prevent Alzheimer’s disease and metabolic syndrome.

## 1. Introduction

The main lipids in milk are fatty acids (FAs), acylglycerols, and cholesterol [1]. Milk fat contains a small quantity of odd and branched chain fatty acids (OBCFAs). However, OBCFAs appear to be differentially accumulated in adipose tissue and milk of cows [2], goats [3], and sheep [4]. The content of these FAs in milk originated from metabolites synthesized by ruminal bacterial, with a large variation in their FAs profiles [5]. Amylolytic bacteria produce more linear odd chain and anteiso FAs than iso FAs, whereas cellulolytic bacteria produce more iso FAs [6]. The biosynthesis of OBCFAs in the rumen is the primary source of milk from ruminant animals [7]. The characteristic of FAs in the ruminal bacteria is largely composed by OBCFAs in the membrane lipids (C15:0; anteiso C15:0; iso C15:0; C17:0; iso C17:0; C17:1 and anteiso C17:0) [8].

OBCFAs have been used as a marker of ruminal bacterial colonization following consuming of fresh herbs [9,10]. Furthermore, some studies have shown that OBCFAs could be used as markers for quantifying ruminal bacteria [11]. Linear odd chain FAs (C15:0 and C17:0) have been used as biomarkers to identify the link between dairy product consumption and disease outcomes [12,13].

Milk contains essential nutrients that are beneficial to human health, e.g., liposoluble vitamins, carotenoids, calcium, bioactive peptides, essential FAs, and sphingolipids [14]. However, cholesterol, saturated FAs, and trans FAs have been associated with increased risk of type II diabetes, obesity, and cardiovascular diseases, which has prompted health authorities to recommend a low consumption of dairy products. As a result, the consumption of OBCFAs is low [15]. Nevertheless, there is an increasing interest of milk OBCFAs, following research reported that several OBCFAs have potential health benefits in humans [16,17]. Recently, it has been reported that during early life Branched-chain fatty acids (BCFAs) play a role in human gut health [18].

OBCFAs are present in small quantities in several vegetables that are incorporated in feedstuff [19]. Some studies have reported that <100 g of OBCFAs per kg of milk can be obtained from feeding, even though all dietary OBCFAs are transferred into milk [20]. This review discusses the importance and origin of milk OBCFAs and the dietary factors that affect OBCFAs biosynthesis in dairy cows. 

## 2. Origin of Milk OBCFAs in Dairy Cows

Decades ago, OCFAs had little significance due to their low physiological concentrations compared to non-OCFAs [21]. In the human body, a large part of OCFAs undergo β-oxidation [22]. While the β-oxidation of OCFAs results in propionyl-CoA, the β-oxidation of even-chain FAs results in cetyl-CoA [17]. Studies have reported that OCFAs formation may occur in the human body via α-oxidation [23]. Furthermore, propionate derived from intestinal bacteria can be used to produce OCFAs in the liver [24].

Milk fat contains a small quantity of OBCFAs. The major OBCFAs in milk are C15:0, C17:0, iso C13:0, iso C14:0, iso C15:0, iso C16:0, iso C17:0, anteiso C15:0, and anteiso-C17:0 [25]. Most of these OBCFAs originate from ruminal bacteria [7]. However, the profile of OBCFAs in milk does not closely match the profile of OBCFAs in ruminal bacteria. The difference between these profiles suggests that a small amount of OBCFAs may originate from post ruminal synthesis [26]. 

The profile of OBCFAs in ruminal bacteria is primarily determined through the enzymes that catalyze FAs’ synthesis in microorganisms, rather than the availability of the precursors [27]. As a result, iso-FAs are abundant in the solid phase of cellulolytic bacteria, while anteiso C15:0 is abundant in the liquid phase of bacteria involved in sugar and pectin fermentation [28]. Bacterial membrane lipids are the main source of OBCFAs in the rumen [29]. In bacteria, de novo synthesis of OBCFAs can proceed systematically. OCFAs can be synthesized via the valerate or propionate elongation pathways [6], with propionyl-CoA rather of acetyl-CoA as the precursor [8]. In addition, contents of OCFAs are higher in milk than in plasma [30], indicating that some OCFAs and anteiso FAs are produced in mammary glands [31]. Even though de novo synthesis of linear OCFAs in mammary glands are not significant in milk [32], several studies have reported that some linear OCFAs such as C15:0 and C17:0 are synthesized from propionate in mammary glands and adipose tissue [33,34]. Propionyl-CoA can serve as the precursor for the synthesis of OCFAs [35]. Precursors of BCFAs are valine, leucine, and isoleucine, which are branched chain amino acids such as 2-methyl butyric, isobutyric, and isovaleric acids [6]. Propionate may be indirectly used in the synthesis of some BCFAs by incorporating methylmalonyl-CoA into the carboxylation product [36]. As a result, a single change in the production of BCFAs or linear-chain FAs is at the specific precursor or product level [8]. 

When cows are supplemented with calcium soap and mixed animal/vegetable fats, 70% of dietary FAs are recovered in the small intestine, of which 106 g/d is derived from the rumen regardless of diet. These FAs are largely OBCFAs, and more than 90% of FA with <14 carbons disappear [37]. Some FAs that are not present in the diet appear in the duodenal digesta, and they are either branched (e.g., C15:0 and C16:0) or odd-numbered carbon chains (C15:0 and C17:0); therefore, they are unique to the ruminal bacteria [38]. 

Milk OBCFAs may originate from (1) ruminal bacteria that produce OBCFAs, which are subsequently transferred to milk or (2) de novo synthesis in mammary glands. 

**Figure 1:**
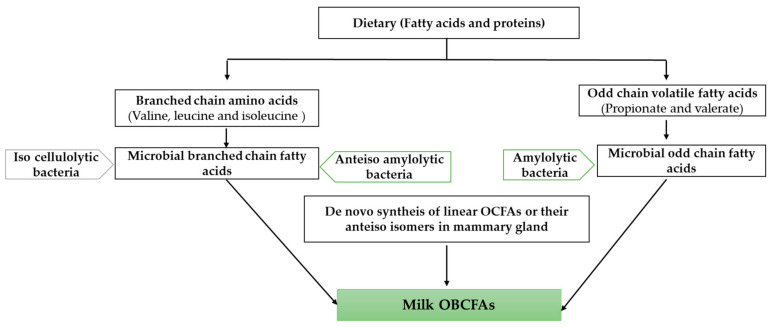
Origin of milk odd and branched chain fatty acids.

Figure 1 provides an illustration of the origin of milk OBCFAs in dairy cows. 

## 3. Dietary Factors Influencing Milk OBCFAs

The nutritional quality of feed influences milk yield and quality in dairy cows [39]. The profile of milk FAs is largely affected by dietary lipid composition and FAs metabolism in rumen [40,41] and mammary glands [42].

Seed, vegetable, and fish oils contribute to an optimal milk composition [43,44]. When cows are fed linseed or flaxseed oil, the ruminal biohydrogenation of cis-9, cis-12, cis-15 C18:3 generates a high amount of FAs isomers [45]. For example, dietary supplements containing flaxseed increase the content of C18:0, C18:1 9c, C18:1 9t, and C18:2 9c 12c in the rumen without significant changes in the content of cis-9, cis-12, cis-15 18:3 and induce the disappearance of the corresponding OCFAs and anteiso FAs [46]. In lactating cows, ground linseed does not affect milk yield or composition, but increases n-3 FAs and decreases OCFAs with ≤16 carbons [47]. The quantity of ground flaxseed in the supplement is negatively associated with OBCFAs in milk and butyrate and acetate in the rumen and positively associated with propionate in the rumen [48]. Dietary FAs composition may affect FAs metabolism in the rumen. Supplementing fat rich in saturated FAs increases the content of (Saturated Fatty Acids) SFAs in milk [49].

Even though they affect ruminal microbial growth and reduce de novo synthesis of microbial FAs, dietary lipids increase FAs uptake in mammary glands [50,51]. Dietary lipids affect the uptake of individual FAs by ruminal bacteria, resulting in a reduction in FAs de novo synthesis by bacteria, which alters the profile of milk FAs [52]. In addition, lipids may inhibit lipogenesis in the mammary glands, likely mediated via the (Conjugated linoleic acids) CLA isomer 18:2 tans 10, cis-12 that is synthesized in the rumen [53]. 

Derived metabolites of volatile FAs (VFAs), such as butyrate, propionate, and acetate, may be used for FAs de novo synthesis by ruminal bacteria. Therefore, these primary end products of ruminal fermentation can affect FAs metabolism in the rumen and mammary glands. Infusing propionate into the rumen increases concentrations of propionate in blood and C15:0 and C17:0 in milk, but not in the rumen [54]. According to Bauman et al. [50] milk C17:0 and cis-9 C17:1 concentrations and acetate-to-propionate ratios are not associated with propionate concentrations in the rumen due to the presence of C17:0 and cis-9 C17:1 in the diet. Fievez et al. [5] reported that there is a positive association between iso FAs and linear OCFAs in milk and acetate and propionate in the rumen. The authors observed that some OBCFAs in milk were associated with the enrichment and relative activities of ruminal bacteria that synthesize OBCFAs. This means that OCFAs content in milk may provide information on specific ruminal conditions [25]. Therefore, the profile of milk OBCFAs might be used as a potential non-invasive method to reveal characteristics of ruminal function [5]. According to Cívico et al. [55] OBCFAs from dairy goats are associated with dietary composition of FAs and may be explored as potential biomarkers in the rumen fermentation. Due to the post-ruminal modifications of OBCFAs, caution should be exercised when using milk OBCFAs to assess ruminal VFAs [26]. 

Even though yield of C15:0, C17:0, iso C15:0, iso C17:0, anteiso-C15:0, and anteiso-C17:0 in milk is related to their duodenal content [56], yield is higher in milk than in the duodenum [20]. 

The modification of milk BCFAs by manipulating dietary fats is not conclusive [50,51,52,53,54,55,56,57]. In general, fat supplementation affects ruminal microbial populations [58] and FAs in milk [50]. For example, addition of unsaturated FAs to the diet reduces saturated FAs in bovine milk, whereas increased unsaturated FAs in dairy cow milk and increased concentrations of dietary 18:3n-3 and 18:2n-6 could effectively increase milk content of cis-9, trans-11 CLA in dairy cow [59,60,61,62,63] while lowering OBCFAs proportions in milk [25,26,27,28,29,30,31,32,33,34,35,36,37,38,39,40,41,42,43,44,45,46,47,48,49,50,51,52,53,54,55,56,57,58,59,60,61,62,63,64]. In contrast, fish oil or microalgae supplementation increases milk OBCFAs concentrations in dairy cows [65,66]. Infusion of branched chain VFAs in the rumen did not affect linear odd-chain FAs, odd anteiso FAs or odd iso FAs in ruminal liquid, ruminal solid, or milk fat. Nevertheless, milk OBCFAs, particularly C13:0, iso C15:0, C17:1 and iso C17:0, varied slightly [54]. 

The type of dietary fat may affect the FAs profile in milk. Substitution of fat abundant in C16:0 FAs with fat abundant in C18:2n-6 FAs at 30 g/kg of DM (Dry Matter) feed in low forage diet reduced the yield of anteiso 13:0 FAs and anteiso 15:0 FAs in milk; however, mixing both types of fat at equal proportions (each at 15 g/kg of DM feed) increased C14:0 iso and C16:0 iso FAs yield in milk [16]. In addition, long-chain PUFAs (Polyunsaturated fatty acids), such as cis-9, cis-12 C18:2, have a toxic effect on ruminal cellulolytic bacteria [67]. Replacing prilled palm fat with sunflower oil linearly reduced the concentration and yield of C13:0 anteiso with non-significant effects on the yield of C15:0 anteiso and total anteiso FAs in milk [16]. These results are probably attributed to the reduction of amylolytic bacteria in the rumen, which are enriched with anteiso FAs according to [5]. Therefore, supplementation with PUFAs may reduce the amount of milk FAs of bacterial origin. 

Dietary type and green feeds influence FAs in milk. In grass silage diet, inclusion of vegetable oils abundant in 18:2n-6 decreased the proportions of numerous iso and anteiso FAs in milk [68]. However, those results were not observed when corn silage and grass silage were mixed in the diet [69]. BCFAs, particularly iso FAs in milk, can be enriched by increasing dietary forage in the diet [16,60,70]. Increasing high-quality grass silage in the diet from 50% to 85% resulted in an increase in linear OCFAs, iso C15:0, and total OBCFAs in milk [70]. A substitution of grass silage with corn silage in the diet increases starch and reduces neutral detergent fiber (NDF), resulting in changes in ruminal pH, microbial populations, VFAs production [71,72], and possibly FAs in the rumen. Patel et al. [70] demonstrated that milk OBCFAs are positively associated with NDF amount in the diet. Morales-Almaráz et al. [73] reported that forage and pasture in the diet increase FAs content in milk. However, an increase in dietary concentrate reduces the efficiency of transit of iso- and anteiso- C15:0 from the duodenal digest to milk. Replacing wheat with corn in the diet reduces bacterial BCFAs content and even-chain saturated FAs in the rumen [46]. According to Bougouin et al. [74] diets containing starch from wheat and maize grain increases milk concentration of various OBCFAs (e.g., C5:0, C7:0, iso C15:0, anteiso C15:0, and anteiso C17:0) to a greater extent than diets containing saturated FAs, extruded rapeseeds, and extruded sunflower seeds. The red clover silage incorporation in dairy cows’ diets increases the amounts of OBCFAs in milk fat [75]. Therefore, OBCFAs in the rumen and milk can be affected by the amount and type of lipids in the diet, forage-to-concentrate ratio, and forage type and proportion in the diet (Table 1, Table 2 and Table 3). 

## 4. Milk OBCFAs and Human Health

Ruminant products are the main source of OBCFAs in the human diet. OBCFAs are produced by ruminal bacteria [8]. The first scientific findings on the negative effects of animal fats on human health generated considerable interest on the chemical composition of these fats. Additionally, it prompted health authorities to recommend a low consumption of dairy products [83]. 

More than 150 different diseases are associated with high dietary lipids, including type II diabetes [84,85], high blood pressure and artery plaque formation [86], obesity [87], neurological disturbances [88], and certain cancers [89,90]. There is a positive association between dairy fat consumption and plasma saturated FAs; therefore, high consumption of dairy fat might be associated with increased risk of cardiovascular diseases. Further studies have shown that these are the ECFAs (Even Chain Fatty Acids) which are related to type 2 diabetes, inflammation and heart disease [91,92,93]. However, Kim and Je [94] reported that dairy intake was negatively correlated with metabolic syndrome, and numerous research showed that there is an association between higher dietary consumption of full-fat dairy and lessen the incidence of cardiovascular disease and type 2 diabetes [95,96,97]. Even though, some previous studies oppose this hypothesis [98].

There is not enough evidence on the link between odd chain FAs (OCFAs) and heart disease and metabolic syndrome. The evidence suggests that OCFAs might have protective effects. Yu and Hu [99] reported a non-significant correlation between C15:0 consumption and heart disease and metabolic disorders. Similar results have been reported by Yakoob et al. [100] and Santaren et al. [101]. The evidence shows non-significant inverse associations OCFAs consumption and atherosclerosis [102,103] and between C15:0 and C17:0 consumption and diabetes [99]. The intake of C15:0 as an active fatty acid diet reduced in vivo anemia, inflammation, fibrosis and dyslipidemia, by mending the function of mitochondria [104]. OCFAs in diet were related to decrease the risk of chronic inflammation, adiposity, cardiovascular disease, type 2 diabetes, metabolic syndrome, nonalcoholic steatohepatitis (NASH), pancreatic cancer and chronic obstructive pulmonary disease in human [91,92,93,94,95,96,97,98,99,100,101,102,103,104,105,106,107,108,109,110,111,112,113]. A meta-analysis of 29 studies discussed the protective effects of OCFAs and of very-long even chain saturated FAs on type II diabetes [106]. OCFAs (C15:0 and C17:0) are significantly and inversely correlated with arterial stiffness and may be negatively correlated with atherosclerosis [114,115]. Tissue levels of OCFAs are lower in patients with Alzheimer’s than in healthy controls [116]. Furthermore, OCFAs might have anti-carcinogenic properties [6]. There are inverse associations between OCFAs consumption and prediabetes and type II diabetes [117,118], cardiovascular disease [119], and insulin resistance [120]. OCFAs increase biotin levels in deficient cases [121] and in cases of peroxisomal disorders [122] and improves cell membrane fluidity [123]. In addition, OCFAs may be used in the treatment of disorders linked to propionate, methylmalonic, and biotin [124].

More than 4% of the total FAs in milk are branched chain FAs (BCFAs) [6]. Dairy and meat from ruminants are the main sources of BCFAs [125]. The importance of BCFAs is attributed to its anticancer activity [6], including on breast cancer cells [126]. Iso C15 has anti-tumor effects on lymphomatoid tumors [66], decreases intestinal necrosis in neonates [127], plays a role in cancer cell death [128], and enhances pancreatic β-cell function [129]. In addition, BCFAs prevent FAs synthesis in tumor cells [130], which rely more on FAs biosynthesis than healthy cells [131].

The recent evidence of large and well-controlled research, meta-analyses and reviews showed that dairy full-fat do not increase cardiometabolic disease risk and may have protective effect against type 2 diabetes and cardiovascular disease [132,133].

## 5. Conclusions

The milk profile of OBCFAs is affected by dietary FAs intake, FAs metabolism in the rumen and mammary glands, and lipid mobilization in the body. Forage and silage in dairy cows’ diets are important in an increasing the amounts of milk OBCFAs. Ruminant products are the main source of OBCFAs in the human diet. OBCFAs have a protective effect on diabetes, Alzheimer’s disease, certain cancers, cardiovascular disease, and atherosclerosis. Understanding the origin of OBCFAs in milk and manipulating the diet of dairy cows to produce OBCFAs-enriched milk can be of scientific and industrial significance.

## Figures and Tables

**Table 1:** Summary of the effects of lipid supplements and forage proportions on OBCFAs synthesis in the rumen of dairy cows.

References	Amounts of Lipid Supplements or Forage Ratio	Animal Breed	Observed Effects on Rumen OBCFAs
[46]	10% of DM of **LW**(extruded linseed and wheat) or LC (extruded linseed and corn)	Holstein cows	**LW**: OCFAs↓, iso FA↑, anteiso FA↓. **LC**: OBCFAs ↓.
[76]	4% **RSO** (rubber seed oil), 4% **FSO** or **RFO** (rubber seed oil + flaxseed oil)	Holstein cows	**RSO**: C15:0↓, C17:0↓. **FSO**: C15:0↓, C17:0↓. **RFO**: C15:0↓, C17:0↓.
[77]	**30:70**, **50:50** and **70:30** forage: concentrate ratio (F:C)	Holstein cows	**70:30:** C11:0↑, C13:0↑, iso C15:0↑, iso C16:0↑, iso C17:0↑ and C17:0↑ **70:30:** anteiso C15:0↓, C15:0↓and total OBCFAs↓
[54]	Infusion of 18.8 mol of **AC** (acetate), **PR** (propionate), **IV** (isovalerate) and **AIV** (anteisovalerate)	Holstein cows	**AIV**: iso C15:0↑ and C17:0↑ in rumen liquid **AIV**: anteiso C15:0↑ and anteiso C17:0↑ in rumen solid **IV**: iso C15:0↑ in rumen solids

**AC**: acetate; **AIV**: anteisovalerate; **Decrease**: (↓); **DM**: Dry matter; **FA**: fatty acids; **F:C**: forage: concentrate ratio; **FSO**: flaxseed oil; **Increase**: (↑); **IV**: isovalerate; **LC**: extruded linseed and corn; **LW**: extruded linseed and wheat; **LW**: OCFAs↓ = LW decrease OCFAs; **No effect**: (↔); **OCFAs**: odd chain fatty acids; **PR**: propionate; **RFO**: rubber seed oil + flaxseed oil; **RSO**: rubber seed oil.

**Table 2:** Summary of the effects of lipid supplements on milk OBCFAs in dairy cows.

References	Concentrationsor Amounts of Lipid Supplement	Animal Breeds	Observed Effects on Milk OBCFAs
[47]	**GFS** (Ground flaxseed) 10% of TMR (total mixed ration)	Jersey cows	11:0↓, 13:0↓, 15:0↓, 17:0↓, iso 14:0↓, iso 15:0↓, anteiso15:0↓, iso 16:0↓, iso 17:0↑, anteiso 17:0↓ (ΣOBCFAs↓).
[78]	2.9% sodium **AC** (acetate) and 2.5% calcium **BU** (butyrate) in a diet.	Holstein cows	Acetate: ΣOBCFAs↓. Butyrate: ΣOBCFAs (↔).
[79]	22 g oil/kg diet **DM** (Dry matter) of **EL** (Extruded linseed), **CPLO** (calcium salts of palm and linseed) or **MR** (milled rapeseed)	Holstein Friesian cows	**EL**: 13:0↓, iso13:0↔, anteiso13:0↓, iso14:0↓, 15:0↓, anteiso15:0↓, iso16:0↓, 17:0↓, iso17:0↑, iso18:0↑. **CPLO**: 13:0↓, iso13:0↔, anteiso13:0↓, iso14:0↓, 15:0↓, anteiso15:0↓, iso16:0↓, 17:0↓, iso17:0↓, iso18:0↑. **MR**: 13:0↓, iso13:0↔, anteiso13:0↓, iso14:0↔, 15:0↓, anteiso15:0↓, iso16:0↓, 17:0↓, iso17:0↓, iso18:0↑.
[16]	30 g/kg of Prilled palm fat (**PPF**)/+ Sunflower oil (**SO**)	Holstein cows	**SO**: anteiso13:0↓, anteiso15:0↓, 15:0↓, 17:0↓, cis-9 15:1↓, and cis917:1↓; **PPF+SO**: iso14:0↑ and iso16:0↑
[80]	30 g/day of LO: linseed oil (**S/LO:** high starch plus linseed oil and **F/LO:** high non-forage plus linseed oil treatments).	Malagueña goats	**S/LO**: Total odd↑, Total iso↓, Total anteiso↑. **F/LO**: Total odd↓, Total iso↓, Total anteiso↓.
[69]	2% of Soybean oil (**SBO**)	Holstein cows	iso 13:0↑, 11:0↓, anteiso 13:0↔, 13:0↓, iso 14:0↓, iso 15:0↓, anteiso 15:0↓, 15:0↓, iso 16:0↑, iso 17:0↑, anteiso 17:0↓, 17:0↓, cis-7 17:0↓, cis-8 17:1↓, cis-9 17:1↓, iso 18:0↓, 19:0↓.
[69]	2% **SBO** (Soybean oil) +1.5% Potassium carbonate (**K2CO3**)	Holstein cows	iso 13:0↑, 11:0↓, anteiso 13:0↓, 13:0↓, iso 14:0↓, iso 15:0↓, anteiso 15:0↓, 15:0↓, iso 16:0↓, iso 17:0↓, anteiso 17:0↑, 17:0↓, cis-7 17:0↓, cis-8 17:1↓, cis-9 17:1↓, iso 18:0↓, 19:0↑.
[50]	450 g/d of **CTL** (lipid free emulsion medium injected into the rumen), **RSO** (lipid free emulsion medium injected into the rumen), **RSF** (saturated fatty acids injected into the rumen), **ASF** (saturated fatty acids injected into the abomasum)	Holstein cows	**RSO**: OCFAs↓, ECisoFAs↔ **RSF**: 17:0+cis-9 17:1↑ **RSF** and ASF: OBCFAs ↔
[48]	0, 5, 10 and 15% of **GFS** (Ground flaxseed)	Jersey cows	**GFS**: OBCFAs↓ linearly
[54]	An Infusion of 18.8 mol of **AC** (acetate), **PR** (propionate), **IV** (isovalerate) and **AIV** (anteisovalerate)	Holstein cows	**PR**: C15:0↑ and C17:0↑; **IV**: iso C15:0↑; **AIV**: C15:0↑
[68]	29g/kg of Plant oils	Ayrshire cows	OBCFAs↓

**AC**: acetate (eg: **Acetate: ΣOBCFAs↓** = Acetate decrease the **sum** of **OBCFAs)**; **AIV**: anteisovalerate; **ASF**: saturated fatty acids injected into the abomasum; **BU**: butyrate; **CTL**: lipid free emulsion medium injected into the rumen; **Decrease**: (↓); **DM**: dry matter; **ECFAs**: even chain fatty acids; **EL:** Extruded linseed **; F/LO**: high non-forage plus linseed oil; **GFS:** Ground flaxseed; **Increase**: (↑); **IV**: isovalerate; **limited effect** or **no effect**: (↔); **LO**: linseed oil; **PPF:** Prilled palm fat; **PR**: propionate; **RSF**: saturated fatty acids injected into the rumen; **RSO**: soybean oil injected into the rumen; **S/LO**: high starch plus linseed oil; **SBO:** Soybean oil) **; SO:** Sunflower oil; **TMR**: Total mixed ration; **Vegetable oils**: sunflower seed oil, rapeseed oil, camelina seed oil or camelina expeller.

**Table 3:** Summary of the effects of proportion, type of forage, and forage-to-concentrate ratio on milk OBCFAs in ruminants.

Reference	Type or Amount of Forage in g/Kg or %	Species or Breed of Animal	Observed Effects on Milk OBCFAs
[81]	**IA** (incremental amount) of **FMH** (Flemingia macrophylla hay): 0, 80, 160, 240 and 320 g kg-1 DM (dry matter)	Saanen x Boer goats	**80**: Σ OBCFAs↓ **160**: Σ OBCFAs↑, **240**: Σ OBCFAs↓, **320**: ΣOBCFAs↑
[16]	**F:C** (forage: concentrate ratio) 39:61, 44:56, or 48:52	Holstein cows	Forage: OBCFAs↑
[82]	A 0.5 ha paddock of **CSP** and two 0.25 ha paddocks 22.4 kg/ha with **PM**	Holstein cows	PM: OBCFAs↑
[70]	With incremental amount of grass silage: 50, 70 and 85%	The Swedish Red Breed of cows	C15:0↑, C17:0↑, iso C15:0↑ and total OBCFAs↑

**CSP**: cool season pasture; **Decrease**: (↓); **DM**: dry matter; **F:C**: forage: concentrate ratio; **FMH**: Flemingia macrophylla hay; **IA**: incremental amount; **Increase**: (↑); **No effect**: (↔); **PM**: warm-season monoculture of pearl millet.
